# Visual “Scrollytelling”: Mapping Aquatic Selfie-Related Incidents in Australia

**DOI:** 10.2196/53067

**Published:** 2024-05-23

**Authors:** Samuel Cornell, Amy E Peden

**Affiliations:** 1 School of Population Health University of New South Wales Sydney Australia

**Keywords:** selfie, map, social media, selfies, scrollama, JavaScript, scrollytelling, Mapbox, incidence, incidents, incident, fatality, fatalities, injury, injuries, retrieval, prevalence, image, images, photo, photos, photograph, photographs, prevalence, Australia, emergency, visualization, visualizations, interactive, location, geography, geographic, geographical, spatial, artificial intelligence, longitude, latitude, visual representation, visual representations

## Introduction

Selfies are a modern, yet preventable cause of injury and death [1]. Medical responses and retrieval of persons, often in challenging terrain, burdens emergency medicine practitioners. To help prevent this issue, this study aimed to visualize selfie-related incidents globally by initially creating a scrollable visual story overlayed on a satellite map of the incidents in Australia. This type of visual storytelling technique using a world map helps illustrate the spatial context of this public health issue.

## Methods

### Overview

Incident data were acquired via publicly accessible news reports and a Wikipedia repository [2] and cleaned and prepared in Excel (Microsoft Corp). Incidents in aquatic areas (eg, coastal locations and inland waterfalls) were included; those in other settings (eg, falls from artificial structures and incidents involving trains) were excluded. Entries for each incident were created using associated media reports, incident types bring falls or drowns. Map coordinates were obtained by locating the incident using Google Maps and inputted into a coordinate finder using the Mapbox Location Helper [3]. Mapbox Studio [4] was used to create a custom map. A satellite template was chosen to best display the geographic context surrounding each selfie incident. The data set was imported into the Mapbox Studio custom map, which populated the data layer onto the map. A heat map setting was chosen for the data. To create the “scrollytelling” map story, the Mapbox storytelling template available from GitHub was used [5]. The primary input is a story comprising sections (chapters), each associated with a particular view of a map, enabling the user to “scroll” down the web page, and the resulting output is a zoomed-in view of a specific case layered on the map. The data and corresponding map visualization are hosted on GitHub and are published with GitHub pages.

### Ethical Considerations

Ethics approval was not required due to the use of publicly available media reports and a Wikipedia repository to create the map.

## Results

**Figure 1:**
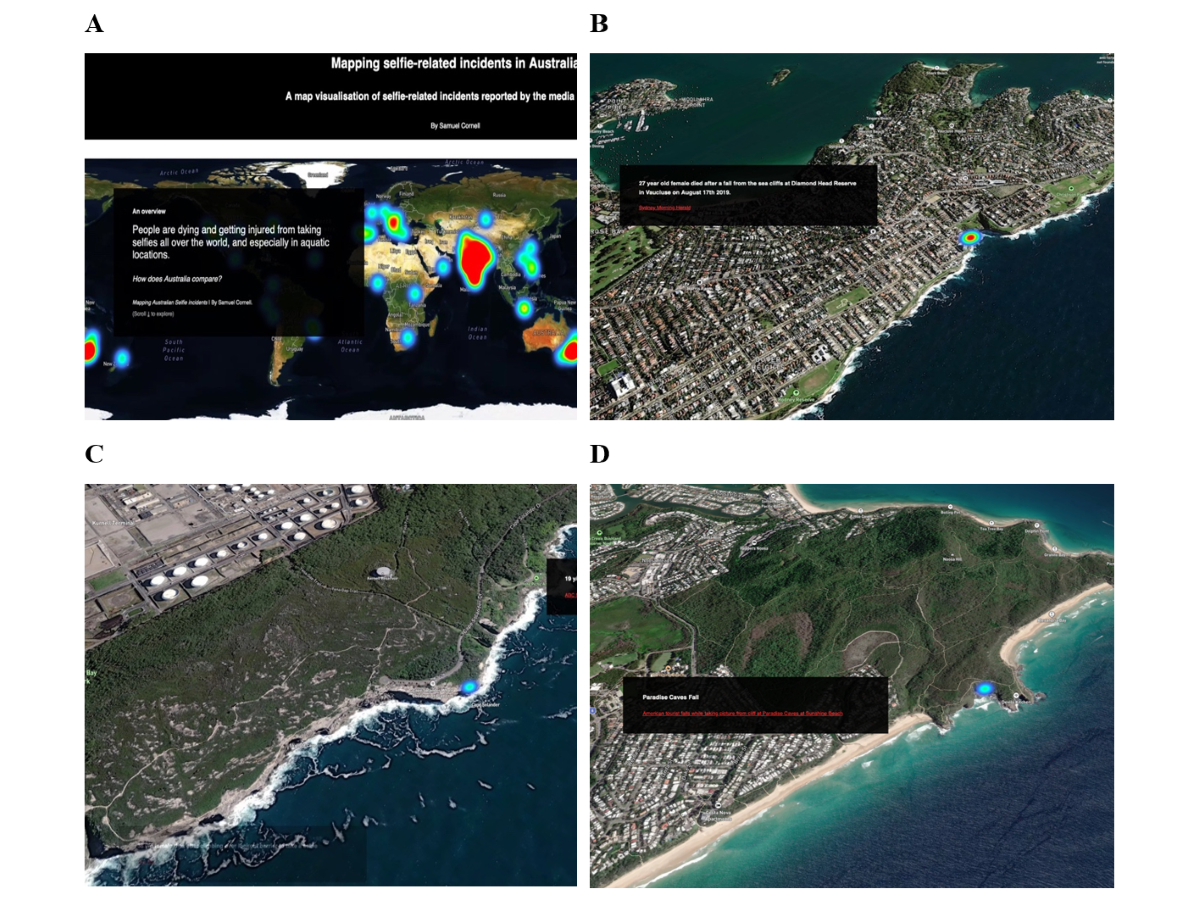
(A) Image taken from the web-based site heat map of worldwide selfie-related incidents. The image provides a worldwide overview of incidents based on the obtained media data used in this study. (B), (C), and (D) Example images acquired from the web-based site. These images illustrate the scrolling story of the heat map focusing on a location that has seen selfie-related incidents. Each incident is indicated in a “chapter,” which provides a description of the incident in that location and a link to the corresponding news report. Images were acquired from Mapbox [7] and OpenStreetMap [8]. OpenStreetMap is licensed under the Open Data Commons Open Database License [9].

The publicly accessible map and data can be viewed on the web [6]. The heat map displays a worldwide overview of 104 cases from June 2014 through August 2023 (Figure 1 [7-9]). Once the user scrolls, the map displays 11 chapters, each showing an individual selfie-related incident in Australia including 9 deaths and 2 serious injuries. All cases reportedly involved emergency services, 3 of which occurred in the same location: Diamond Bay, Vaucluse, a suburb of Sydney, New South Wales. Two cases occurred at Gibraltar Falls, Australian Capital Territory. Further cases in Australia that are included in the map story are detailed in Multimedia Appendix 1.

## Discussion

Our heat map of media-based incident data provides a globally applicable visual representation of the selfie-incident phenomenon in Australia. Using a scrolling story template, overlayed on top of a map, selfie incidents can be illustrated in a geographic context. It is clear from the heat map that certain locations worldwide (nations such as India) and in Australia (cities such as Sydney) require specific and targeted prevention strategies to attenuate the incidence of selfie incidents, which is in line with their specific topographical realities.

Future research in this space should seek to further ascertain the burden on emergency and retrieval services by evaluating response times, resource allocation, retrieval or rescue methods, and health care costs associated with treating selfie-related injuries. Geographic disparities in service usage and response times, terrain, and retrieval or rescue methods should be identified and added to the visual map.

Understanding the geographic distribution and burden of selfie-related incidents is essential for designing targeted public awareness campaigns, improving safety regulations, and optimizing the allocation of resources for emergency and retrieval services.

The main limitation results from the use of media cases as the basis of these incident reports. It is not possible to acquire precise latitude and longitude coordinates. The coordinates provide the best approximation using details provided in media reports and analyzing the geography described in the report. Nevertheless, this map provides a good overview of the geographic nature of selfie-related incidents. Given the ethical limitations regarding the use of coronial data and identification of individual incidents, publicly available information of this nature remains the most appropriate data source.

In conclusion, selfie-related incidents present a significant geographic challenge for emergency services and retrievalists due to the inherent geographic context surrounding this type of event. Mapping selfie events may be a useful method of analyzing and tracking these phenomena and may be of benefit to emergency managers and land managers in collaborating to attenuate this issue.

## Appendix

Multimedia Appendix 1 Table displaying cases used in the scrollytelling map.

## Data Availability

All data are publicly available and can be found in a GitHub repository [10].
