# Supplementary material for: Visual “Scrollytelling”: Mapping Aquatic Selfie-Related Incidents in Australia
Source: Interact J Med Res. 2024 May 23;13:e53067. doi: 10.2196/53067 (PMC11157173; doi:10.2196/53067)
Supplement: Multimedia Appendix 1 [file ijmr_v13i1e53067_app1.docx]

Table 1.

| **Date of incident** | **Country** | **Latitude** | **Longitude** | **Type of Incident** | **Death or injury** | **Description** | **Media report URL** |
| --- | --- | --- | --- | --- | --- | --- | --- |
| 19-May-18 | Australia | -35.11867 | 117.89275 | Fall | Death | A student fell to his death from a cliff while taking photos at a popular tourist attraction in Australia.  The 20-year-old was reportedly posing for selfies with friends when he slipped off the 40-metre precipice at The Gap, near the historic port town of Albany. | <https://www.independent.co.uk/news/world/australasia/student-selfie-fall-death-cliff-gap-albany-australia-a8359546.html> |
| 22-Jul-18 | Australia | -34.016615 | 151.231784 | Fall | Death | A 19-year-old man from West Haven, Utah, fell to his death at Cape Solander in Kurnell near Sydney, Australia. Slipped while taking photos. The local Mayor said police had recovered a mobile phone with photographs of the teenager taken moments before he fell. | <https://www.abc.net.au/news/2018-07-24/kurnell-cliff-fall-mormon-missionary-gavin-zimmerman/10029406> |
| 28-Feb-19 | Australia | -34.19481 | 151.03861 | Fall | Death | 22-year-old man washed off the rocks on Monday has been called off. The incident occurred at the peak of high tide with the NPWS website listing the wave risk as 'extreme'. | <https://www.abc.net.au/news/2019-02-28/call-to-close-access-to-social-media-favourite-figure-8-pools/10853854> |
| 17-Aug-19 | Australia | -33.862684 | 151.282771 | Fall | Death | A 27-year-old woman died after falling from cliff at one of Sydney's most popular selfie spots. | <https://www.theguardian.com/australia-news/2019/aug/17/sydney-cliff-fatality-woman-falls-to-death-at-popular-selfie-spot> |
| 12-Jan-20 | Australia | -33.862684 | 151.282771 | Fall | Death | A 21-year-old woman from Lincoln, England, fell from a 98-foot-high cliff in Diamond Bay Reserve in Sydney, Australia. According to police, the woman accompanied by seven friends, climbed a fence to sit on the edge of the cliff and take selfies. The Diamond Bay Reserve is known as a tourist attraction. | <https://www.9news.com.au/national/vaucluse-diamond-bay-woman-dies-falling-off-cliff-popular-coastal-selfie-spot/b2cef065-df6f-4712-8217-ecb183313de8> |
| 17/4/2020 | Australia | -33.862684 | 151.282771 | Fall | Injury | 15-year-old fells at Vaucluse whilst taking selfie. Survived with multiple injuries. | <https://www.9news.com.au/national/teenage-girl-falls-from-deadly-selfie-spot-diamond-bay-reserve-sydney-vaucluse/5874baa1-fc17-4539-b694-12cb557bb3bb> |
| 12-Dec-20 | Australia | -37.239176 | 142.410725 | Fall | Death | A 38-year-old woman from North Victoria fell 80 meters and died on scene at The Grampians in Victoria at a popular tourist photo spot. According to local authorities, she jumped over a safety barrier and tripped off the cliff edge. | <https://www.abc.net.au/news/2022-08-02/grampians-death-coronial-inquest-more-warning-signs-needed/101290142> |
| 9-Jul-21 | Australia | -27.47793 | 153.03444 | Fall | Death | 33-year-old woman found dead at the base of the popular Kangaroo Point cliffs in Brisbane may have fallen after losing her balance while taking a selfie. | <https://7news.com.au/news/qld/brazilian-woman-who-fell-from-brisbanes-kangaroo-point-cliffs-may-have-been-taking-selfie-c-3343974> |
| 12-Feb-23 | Australia | -35.4868338 | 148.9345944 | Fall | Death | 19-year-old male was found dead after falling off a cliff at Gibraltar Falls, ACT. | <https://7news.com.au/news/act/19-year-old-man-found-dead-at-gibraltar-falls-identified-c-9772754> |
| 19-Feb-23 | Australia | -35.4868338 | 148.9345944 | Fall | Death | 22-year-old man was found unconscious in the water at Gibraltar Falls, ACT. | <https://7news.com.au/news/act/man-who-died-falling-from-gibraltar-falls-identified-as-22-year-old-patrick-prevett-c-9809482> |
| 25-Aug-23 | Australia | -26.39232 | 153.11678 | Fall | Injury | American tourist falls 5m while taking picture from cliff at Paradise Caves at Sunshine Beach near Noosa | <https://7news.com.au/news/qld/woman-falls-5m-from-cliff-of-paradise-caves-at-sunshine-beach-near-noosa-c-11521583> |
